# Tailoring the Mesoscopic TiO_2_ Layer: Concomitant Parameters for Enabling High-Performance Perovskite Solar Cells

**DOI:** 10.1186/s11671-016-1809-7

**Published:** 2017-01-19

**Authors:** Taehyun Hwang, Sangheon Lee, Jinhyun Kim, Jaewon Kim, Chunjoong Kim, Byungha Shin, Byungwoo Park

**Affiliations:** 1 Department of Materials Science and Engineering, WCU Hybrid Materials Program, Research Institute of Advanced Materials, Seoul National University, Seoul, 08826 Korea; 2 School of Materials Science and Engineering, Chungnam National University, Daejeon, 34134 Korea; 3 Department of Materials Science and Engineering, Korea Advanced Institute of Science and Technology, Daejeon, 34141 Korea

**Keywords:** Perovskite solar cell, Nanostructural engineering, Light management, Modeling, Shunting path

## Abstract

Architectural control over the mesoporous TiO_2_ film, a common electron-transport layer for organic-inorganic hybrid perovskite solar cells, is conducted by employing sub-micron sized polystyrene beads as sacrificial template. Such tailored TiO_2_ layer is shown to induce asymmetric enhancement of light absorption notably in the long-wavelength region with red-shifted absorption onset of perovskite, leading to ~20% increase of photocurrent and ~10% increase of power conversion efficiency. This enhancement is likely to be originated from the enlarged CH_3_NH_3_PbI_3_(Cl) grains residing in the sub-micron pores rather than from the effect of reduced perovskite-TiO_2_ interfacial area, which is supported from optical bandgap change, haze transmission of incident light, and one-diode model parameters correlated with the internal surface area of microporous TiO_2_ layers. With the templating strategy suggested, the necessity of proper hole-blocking method is discussed to prevent any direct contact of the large perovskite grains infiltrated into the intended pores of TiO_2_ scaffold, further mitigating the interfacial recombination and leading to ~20% improvement in power conversion efficiency compared with the control device using conventional solution-processed hole blocking TiO_2_. Thereby, the imperatives that originate from the structural engineering of the electron-transport layer are discussed to understand the governing elements for the improved device performance.

**Electronic supplementary material:**

The online version of this article (doi:10.1186/s11671-016-1809-7) contains supplementary material, which is available to authorized users.

## Background

Organic-inorganic hybrid perovskites (CH_3_NH_3_PbI_3_) have drawn enormous attentions due to their superior optoelectronic properties and versatilities in applications [1, 2]. For photovoltaic devices, many strategies have been attempted to improve the power-conversion efficiency. One among many deals with refining the perovskite film itself to reduce the trap states and unwanted electron-hole recombination. Generally, defects in grains or grain boundaries act as trap sites for the charge carriers and consequently decrease the charge collection efficiency [3–6]. Indeed, much effort aimed at the single-crystal perovskites caused successful results for the high photon-to-charge conversion efficiency [7–10]. Therefore, examining the strategies to control the crystallization for the defect reduction is necessary to achieve better-performing perovskite photovoltaics.

Defect-reduced perovskite films can be realized by directly modifying the perovskite synthesis conditions (e.g., reaction environment, precursor stoichiometry, crystallization atmosphere, etc.) [11–13] or by altering the mesoscopic structure of the underlying layers over which the perovskite film synthesis is conducted. The perovskite films are generally deposited upon mesoscopic scaffolds composed of oxide nanoparticles like TiO_2_, into which the perovskite precursors infiltrate and form small crystallites whose dimensions are defined by the internal pore size of mother scaffold. Enlarging the pores in the scaffold, and hence, increasing the infiltrated perovskite grains is expected to reduce the defects by grain boundaries. At the same time, the internal electric field that is formed at the semiconductor junction may further assist the charge separation. Light trapping by the nanostructural engineering will also yield an additional merit for the performance enhancement [14].

To exploit the potential benefits of large-sized single crystalline perovskite, we herein controlled the nanostructures of mesoscopic TiO_2_ layer to infiltrate the enlarged CH_3_NH_3_PbI_3_(Cl) grains. Introduction of sacrificial templates during photoelectrode fabrication, one of the facile methods to obtain the controlled pore size and internal surface area [15–18], was applied to render sub-micron sized pores where the large perovskite grains can be accommodated. The concomitant effect of perovskite crystallinity, perovskite-TiO_2_ interfacial area, and light trapping was investigated to understand the change of photovoltaic parameters resulted from the templating method. Furthermore, since the templated porous layer with hundred-nanometer large open pores inevitably raises the necessity for the complete compactness of hole-blocking layers against charge recombination at the FTO-perovskite direct contact, an alternative blocking layer was applied, providing additional power-conversion efficiency improvement. The essential issues in nanostructural engineering were discussed with the correlated solar-cell parameters.

## Methods

### Preparation of Polystyrene (PS)-TiO_2_ Mixture Solution

The PS-TiO_2_ mixture solution was prepared by mixing the ethanol-based PS solution (PS microsphere with 200 nm in diameter) and the TiO_2_ paste (anatase-phase TiO_2_ nanoparticles with ~20 nm in diameter) with various ratios (PS/TiO_2_ = 1:10, 1:5, and 1:2 in wt. % ratio). The PS-TiO_2_ solution was then diluted with identical solvent (PS-TiO_2_/ethanol = 2:5 in wt. % ratio) for spin-coating. To prepare the bare TiO_2_ solution without polystyrene for a reference, the TiO_2_ paste was diluted with anhydrous ethanol to the corresponding wt. % ratio.

### CH_3_NH_3_PbI_3_(Cl) Deposition

The PbI_2_ pre-coating was performed following our previous report [19]. The 3:1 molar ratio of MAI/PbCl_2_ in DMF (perovskite precursor solution; 2.64 M of MAI and 0.88 M of PbCl_2_) was then spin-coated at 2000 rpm for 60 s on the PbI_2_ pre-coated layer or the TiO_2_ compact layer and annealed at 100 °C for 50 min. To enhance the coverage and obtain the similar thicknesses of the perovskite capping layers in the PS-templated TiO_2_ cases, spin-coating conditions were optimized. Spin-coating speed was reduced from 6500 to 1500 rpm for PbI_2_ and from 2000 to 1500 rpm for the perovskite precursor solution. The precursor concentration was increased (from molar ratio of 2.64:0.88 to 4.08:1.36 between MAI and PbCl_2_) with the increased annealing time (from 50 to 135 min), and the optimization was checked in the aspect of the perovskite crystallization from diffraction. As a control group, molar ratio of 2.64:0.88 between MAI and PbCl_2_ was also used on the 1:10 PS-templated TiO_2_, and we referred it as “1:10 (unoptimized)” since the perovskite did not fully cover the top of the 1:10 PS-templated TiO_2_. Every perovskite deposition was processed in air.

### Solar Cell Fabrication

A fluorine-doped tin oxide (FTO) substrate was cleaned, and the TiO_2_ compact layer was deposited using the 150 and 300 mM solutions of titanium diisopropoxide bis(acetylacetonate) in 1-butanol through the spin-coating followed by the annealing at 500 °C [20]. Then, the substrate was immersed in a 40 mM TiCl_4_ aqueous solution and treated in 70 °C oven for 30 min, followed by annealing at 500 °C. Bare TiO_2_ or PS/TiO_2_ solution was spin-coated at 2500 rpm for 30 s, and the substrate was annealed at 500 °C to remove the polystyrene templates. Then, TiCl_4_ treatment was performed again, and MAPbI_3_(Cl) layer was deposited as mentioned in the previous paragraph. Hole transport layer was coated using the spiro-OMeTAD solution (72.8 mg in 1 mL of chlorobenzene) with the addition of 17.5 μL of Li-TFSI stock solution (520 mg in 1 mL of acetonitrile) and 28.8 μL of tert-butylpyridine [20]. Finally, Au electrode was thermally evaporated.

The TiO_2_ compact layer was separately prepared by rf-magnetron sputtering as an alternative blocking layer [21], instead of using conventional titanium diisopropoxide bis(acetylacetonate) solution. The deposition was performed using the TiO_2_ target (anatase, 99.99%; 5-cm diameter and 0.6-cm thickness) at room temperature under an Ar atmosphere with the operating pressure of 13 mTorr and rf power of 120 W. Except for the blocking layer deposition, all the other procedures were exactly identical to the solar cell fabrication conditions described above.

### Characterization

The crystal structure was examined by X-ray diffraction (XRD) (D8 Advance: Bruker). The images of secondary electrons and back scattered electrons were collected from field-emission scanning electron microscope (FESEM) (Merlin Compact: Zeiss), with the energy-dispersive X-ray spectroscopy (SEM-EDS). The absorbance and transmittance of the films were recorded through a UV-Vis spectrophotometer (Cary 5000: Agilent Technologies) with the integrating sphere, and the optical bandgap was evaluated from the *α* ^2^ vs. *hν* (photon energy) analysis. Photocurrent density-voltage (*J*-*V*) curves were obtained by the solar cell measurement system (K3000: McScience) with a solar simulator (Xenon lamp, air mass (AM) 1.5 at 100 mW cm^−2^). During the measurement, black mask of 0.09 cm^2^ was applied, and the scan rate was fixed to 150 mV s^−1^ (reverse direction).

## Results and Discussion

**Fig. 1:**
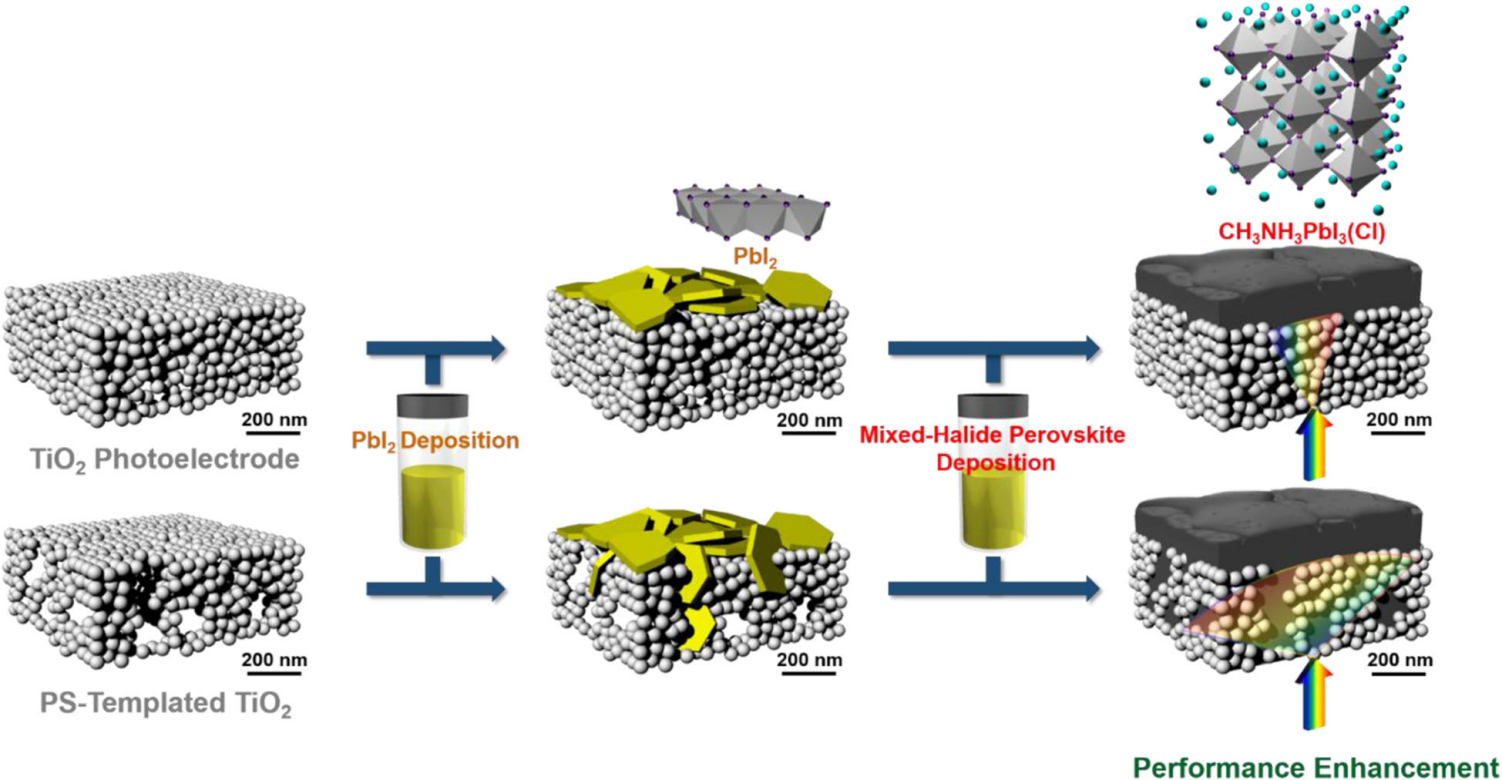
Schematic diagram illustrating the deposition of mixed-halide perovskite on the nanostructure-tailored TiO_2_ scaffold. *Upper row* is the MAPbI_3_(Cl) deposition on the general mesoporous TiO_2_ substrate. *Lower* is similar except for the nanostructural engineering of TiO_2_ using polystyrene (PS) as a sacrificial template. *Yellow crystals* are PbI_2_ consisting of the edge-sharing of PbI_6_ octahedrons, and *black crystals* are mixed-halide perovskites consisting of corner-sharing of PbI_6_ with MA^+^ insertion (*light-blue*)

For the achievement of high photon-to-charge conversion efficiency in solar cell operation, the high light absorption followed by the electron-hole generation and facile separation of carriers into each electrode should be guaranteed throughout the cell structure. Thus, the essential parameters that can affect these phenomena should be considered [22–25]. For high photoresponsivity, the composition and morphology of MAPbI_3_ can be altered to broaden the absorption spectra [26–29]. For the electron-hole pair separation, internal electric field driven from the semiconductor junction can be utilized, and it is supported by the result that the MAPbI_3_ phase forms the depletion region at the interface with TiO_2_ in approximately hundreds of nanometers [30]. Having sufficiently large pores in the scaffold, the size of which is comparable to the depletion layer in the perovskite, therefore shall give a microstructural modification of infiltrated perovskite grains with the size desirable in terms of electron-hole separation. A comprehensive outline for the approach suggested above is given in Fig. 1, depicting the nanostructural engineering of TiO_2_ accompanying the perovskite deposition for the intended large crystal infiltration.

**Fig. 2:**
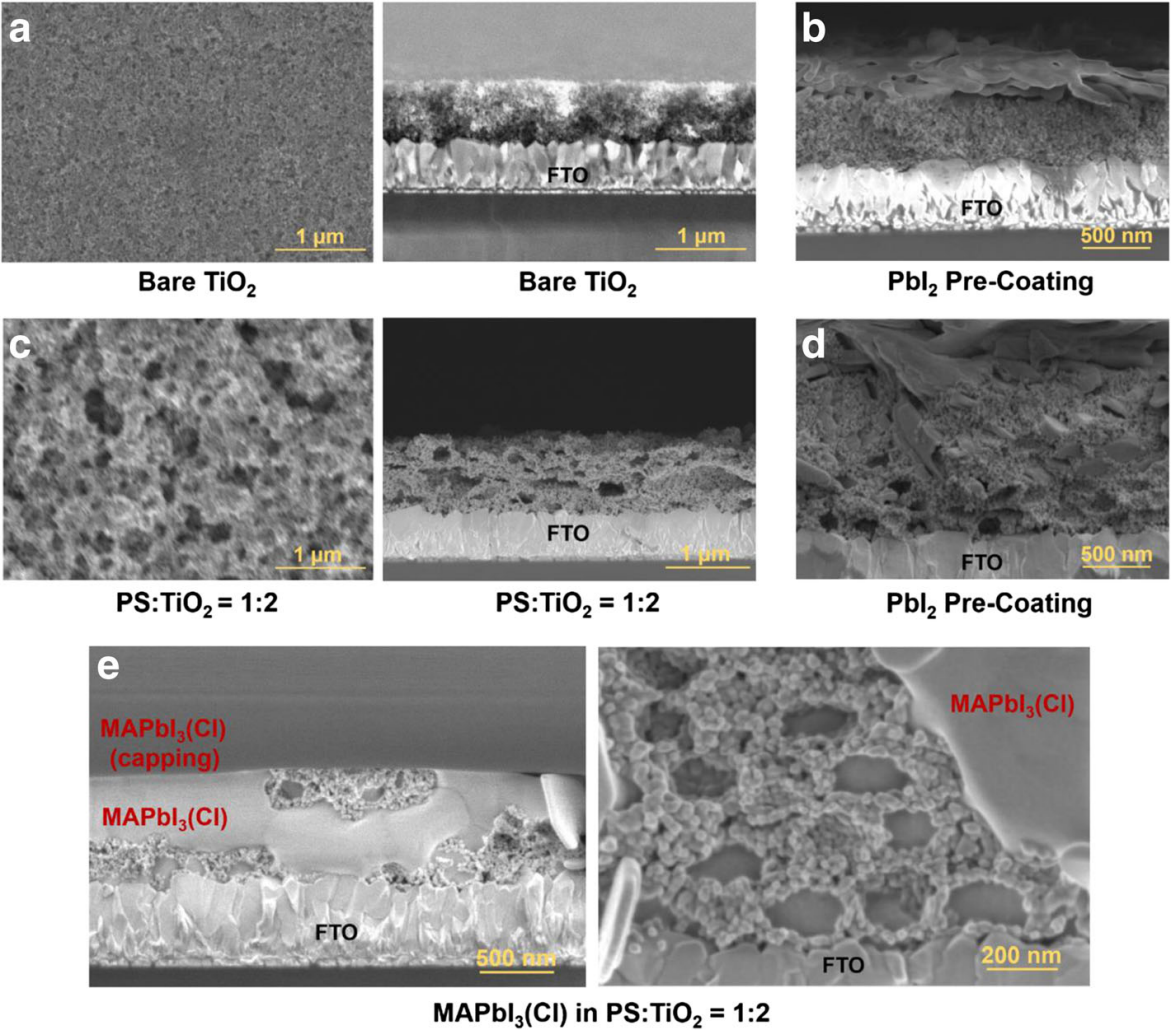
Scanning electron microscopy images showing the TiO_2_ nanostructures with the PbI_2_ pre-coating and MAPbI_3_(Cl) infiltration into the polystyrene-templated TiO_2_ scaffold. **a** Plan and cross-sectional view of bare TiO_2_. **b** PbI_2_-pre-coated bare TiO_2_. **c** Plan and cross-sectional view of TiO_2_ made from the 1:2 wt. % ratio of PS/TiO_2_ (PS/TiO_2_ = 1:2). **d** PbI_2_-pre-coated TiO_2_ from PS/TiO_2_ = 1:2. **e** Cross section of MAPbI_3_(Cl) in TiO_2_ and the magnified view

**Fig. 3:**
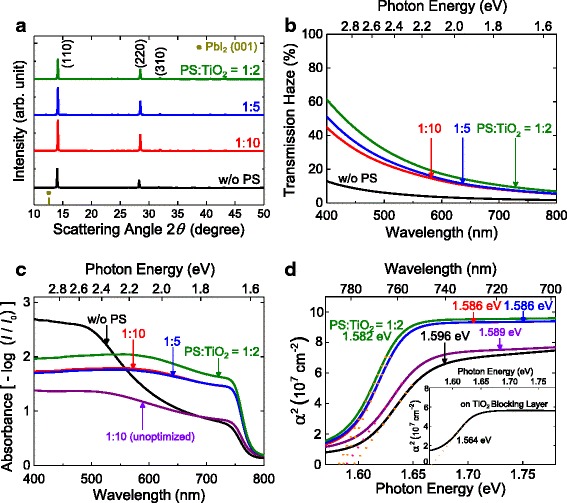
The effect of PS ratio on the nanostructures and optical properties of MAPbI_3_(Cl) perovskite. **a** X-ray diffraction of MAPbI_3_(Cl) films. **b** Transmission haze of TiO_2_ with different ratios of PS bead. **c** Absorbance of MAPbI_3_(Cl) on the corresponding TiO_2_, and **d** the determination of the optical bandgap for the MAPbI_3_(Cl) film. Samples are without PS bead (w/o PS), 1:10 wt. % ratio of PS bead in TiO_2_ paste (PS/TiO_2_ = 1:10 (before and after optimization)), 1:5 wt. % ratio of PS to TiO_2_ (1:5 (optimized)), and 1:2 wt. % ratio (1:2 (optimized)). For comparison, the optical bandgap energy of MAPbI_3_(Cl) film on a compact TiO_2_ is shown in the *inset* of (**d**)

To amend the pore size of TiO_2_ layers and finally to adjust the grain size of infiltrated perovskite, a sacrificial template is facilely incorporated into the commercially available nanoparticle-based TiO_2_ pastes by mixing with sub-micron sized polystyrene (PS) beads, varying the composition from PS/TiO_2_ = 1:10 to 1:2 [15–18]. Rather thick TiO_2_ porous film (~800 nm) is used for solar cell in this case to investigate the effects of interface between perovskite and TiO_2_ on the cell performance [2, 22]. Micropores left after the PS removal are successfully filled with PbI_2_ by the pre-coating step, and the remaining PbI_2_ crystals are stacked on TiO_2_ (Fig. 2a–d and Additional file 1: Figure S1). These pre-coating method guarantees the enlarged grains and crystallinity of the converted perovskite since the original PbI_6_ octahedron in the PbI_2_ structure maintains its framework after the reaction with MA^+^ and I^−^ in the precursor [19]. As shown in Fig. 2e and Additional file 1: Figure S1, layered-PbI_2_ crystals are converted into perovskite, filling the intended ~200-nm micropores. Also, the conversion into MAPbI_3_(Cl) is completed while maintaining the [110] orientation without remnant, as verified from the diffraction in Fig. 3a (magnification in Additional file 1: Figure S2(a)).

The back-scattered electron (BSE) imaging is a useful tool to identify the compositional contrast which originates from the atomic-number difference [31]. The BSE images in Additional file 1: Figure S3 confirm that regular ellipsoidal perovskites are clearly formed in the intended micropores. Furthermore, it is used to confirm the PbI_2_ pre-coating influence on the perovskite infiltration into the mesoporous TiO_2_ layer (mp-TiO_2_) [32]. The PbI_2_ pre-coating indeed do not interfere with the perovskite infiltration into the mp-TiO_2_ (without PS) based on the BSE intensity comparison between Additional file 1: Figure S3(b) and (c). This is further examined by the elemental mapping (SEM-EDS): the distributions of Pb and I are the same whether the PbI_2_ pre-coating is performed or not (Additional file 1: Figure S4(a) and (b)) and whether the TiO_2_ layer is altered by the PS sacrificial template or not (Additional file 1: Figure S4(b) and (c)). The BSE intensity and the EDS mapping confirm that the interfacial area between the perovskite and TiO_2_ is decreased with the increased PS fraction, since the nanoparticulated-TiO_2_ film consisting of ~20 nm-sized-nanoparticle has a larger internal surface than the TiO_2_ film with the intended ~200-nm micropores. The enlarged perovskite grain by PS incorporation is supported accordingly from the above results.

Haze transmission is the ratio of the diffused transmittance to the total transmittance, and discloses the degree of incident light scattering [33]. The PS-templated TiO_2_ looks opaque, and the haze increases as the PS ratio rises (Fig. 3b). Also, asymmetric elevation of absorbance is observed from MAPbI_3_(Cl) with increasing PS ratio as shown in Fig. 3c. This is due to the increased light scattering from TiO_2_ and perovskite by the intended large crystals. In addition, the bandgap of mixed-halide perovskite is red-shifted by ~10 meV from the Tauc plot (Fig. 3d). This optical bandgap change is also observed when the identical mixed-halide precursor solution is used for the bare and 1:10 cases (“unoptimized” which is explained in the experimental section). This red-shift is not from the different quantity of Cl since the (110) peak of MAPbI_3_(Cl) is identical between the bare and 1:10 case (Additional file 1: Figure S2(b)) [34].

When the concentration of mixed-halide solution is increased by ~50% while maintaining the MAI/PbCl_2_ ratio as 3:1 to improve the perovskite coverage for the PS-templated TiO_2_ cases, the (110) peak shifts to the high scattering angle (Additional file 1: Figure S2(a), identical to Fig. 3a with proper magnification). The lattice parameters *a* and *c* in tetragonal (space group I4/m) are changed, respectively, from 0.892 to 0.886 nm and from 1.261 to 1.251 nm. The apparent optical bandgap can vary by the Cl concentration in MAPbI_3_(Cl), Burstein-Moss effect (carrier concentration), quantum confinement effect, and/or grains and grain boundaries [9, 10, 26, 35–37]. The Burstein-Moss and quantum confinement effects are not pertinent to this system considering that the composition of perovskite was confirmed to be the same for all the cases, and the grain size was out of the regime where the quantum confinement effect works in [36, 37]. Therefore, the optical bandgap change is expected to be caused by the increased perovskite crystal sizes and Cl concentrations (based on the tetragonal unit-cell size). To verify the grain-size effect on the absorption shift, MAPbI_3_(Cl) perovskite is deposited with the identical concentration of mixed-halide solution to the bare (without PS) on planar TiO_2_ layer (Additional file 1: Figure S5). From the diffraction and SEM images of MAPbI_3_(Cl) film, MAPbI_3_(Cl) grown on the TiO_2_ planar layer exhibits micrometer-sized lateral grains with the ~30-meV red-shift compared to the bare (without PS) in the absorption onset (inset in Fig. 3d), and this additionally supports the absorption-edge shift with respect to the perovskite grain size.

**Fig. 4:**
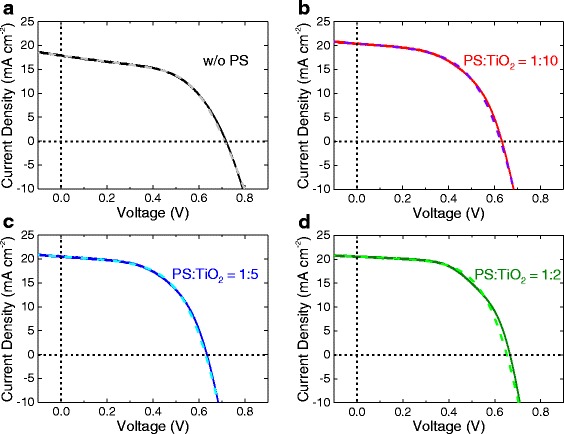
The effect of PS ratio on the performance of the perovskite solar cell. *J*-*V* curve (*solid line*) on **a** bare TiO_2_, **b** 1:10, **c** 1:5, and **d** 1:2 PS-templated TiO_2_ under light exposure (AM 1.5, 100 mW cm^−2^), and the corresponding fitting result (*dashed line*) from the ideal one-diode model (described in the following Additional file 1: Figure S7). The corresponding photovoltaic parameters are summarized in Tables 1 and 2

The MAPbI_3_(Cl) solar cells are fabricated on each PS-templated TiO_2_ with varying PS ratios (Fig. 4 and Additional file 1: Figure S6) to understand the grain size and interfacial effects on the solar cell performance. The best and the average values of short-circuit current density (*J*_sc_), open-circuit voltage (*V*_oc_), fill factor (FF), and power-conversion efficiency (*η*) are summarized in Table 1. For PS/TiO_2_ = 1:10 case, the *η* is rather decreased in spite of ~13% improvement of *J*_sc_, which is due to the inferior *V*_oc_ and FF. Inferior *V*_oc_ in this case should be resolved to overcome the low efficiency, and we have considered several approaches, specifically focusing on the defect reduction that can cause recombination in the perovskite and at the interfaces [38–42]. However, as the PS ratio is increased, these parameters are recovered by ~20 mV and ~4% with the additional increase of *J*_sc_, leading to approximately 10% increase of *η* for the PS/TiO_2_ = 1:2 case compared with the control devices without PS templating.

**Table 1:** The effect of polystyrene (PS) ratio on the perovskite solar cell performance

Substrate	*J*_sc_ [mA cm^−2^]	*V*_oc_ [V]	*FF* [%]	*η* [%]
w/o PS	17.9 (16.8)	0.719 (0.713)	53.4 (52.1)	6.87 (6.24)
1:10	20.5 (18.9)	0.633 (0.621)	52.7 (50.8)	6.84 (5.94)
1:5	20.6 (19.3)	0.635 (0.626)	54.5 (53.9)	7.13 (6.51)
1:2	20.5 (19.7)	0.667 (0.644)	56.6 (54.7)	7.74 (6.93)

Short-circuit current density (*J*_sc_), open-circuit voltage (*V*_oc_), fill factor (*FF*), and power-conversion efficiency (*η*) of perovskite solar cells, without PS and with various ratios of PS-templated TiO_2_. TiO_2_ blocking layer is deposited by solution. (Data in the bracket are the averaged ones from more than four cells in each condition.)

Analyses of *J*-*V* curves based on the one-diode model provide useful parameters helpful to figure out the interfacial effects. The *J*-*V* curves are fitted using the ideal one-diode model described in Additional file 1: Figure S7, and the resultant fit curves are shown with the dashed lines in Fig. 4 with the extracted solar-cell parameters in Table 2 [43, 44]. (Fitting results of *J*-*V* under dark conditions are shown in Additional file 1: Figure S8.) The fit result shows that the dark-saturation current density (*J*_0_) and the ideality factor (*n*) are worsened from ~10^−5^ to ~10^−3^ mA cm^−2^ and ~2 to ~3, respectively, when PS is introduced. The back electron transfer from the FTO front electrode to the perovskite by the ~200-nm penetration may cause the recombination path, as seen in SEM image of Fig. 2e [45].

**Table 2:** Photovoltaic parameters extracted from the ideal one-diode model

Substrate	*J*_0_ [mA cm^−2^]	*J*_*ph*_ [mA cm^−2^]	*R*_*s*_ [Ω cm^2^]	*R*_*sh*_ [Ω cm^2^]	*R*_*sh*_^*recom*^ [Ω cm^2^]	*n*
w/o PS	9.39 × 10^−6^ (± 3.62 × 10^−6^)	18.6 (± 0.1)	5.90 (± 0.19)	152.0 (± 0.8)	152.0 (± 0.8)	1.94 (± 0.05)
1:10	1.92 × 10^−2^ (± 0.25 × 10^−2^)	20.6 (± 0.1)	1.69 (± 0.11)	286.6 (± 5.7)	287.2 (± 5.7)	3.53 (± 0.07)
1:5	7.84 × 10^−3^ (± 1.27 × 10^−3^)	20.7 (± 0.1)	1.95 (± 0.11)	303.6 (± 5.8)	304.7 (± 5.8)	3.13 (± 0.07)
1:2	2.77 × 10^−3^ (± 0.67 × 10^−3^)	20.7 (± 0.1)	2.00 (± 0.16)	440.4 (± 10.6)	441.8 (± 10.7)	2.85 (± 0.09)

Dark-saturation current density (*J*_0_), photogenerated current density (*J*_*ph*_), series resistance (*R*_*s*_), shunt resistance (*R*_*sh*_), recombination shunt resistance (*R*_*sh*_^*recom*^), and ideality factor (*n*), respectively, from the cell of the highest efficiency in each condition (AM 1.5 at 100 mW cm^−2^). (Parameters are described in the following Additional file 1: Figure S7.)

**Fig. 5:**
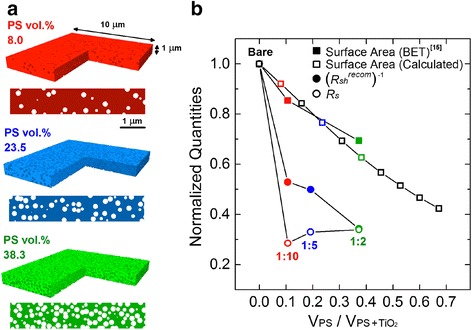
Calculated surface area for the analyses of surface area of the PS-templated TiO_2_ and its comparison with the photovoltaic parameters. **a** Typical simulation results describing the distribution of 200-nm polystyrene-induced pores with the number variation of PS microspheres (PS vol. %) in 10 × 10 × 1 μm^3^, and the corresponding typical cross-sectional views. **b** Polystyrene-induced loss of total surface area (*open squares*), plotted with *R*_*s*_ (series resistance) and *R*_*sh*_^*recom*^ (recombination shunt resistance) from the experimental *J*-*V* curve, as extracted from the ideal one-diode model. (Simulated surface area is also compared to the experimental values from Ref. [15].)

However, the series resistance (*R*_*s*_) is improved from 5.9 to 2.0 Ω cm^2^, and the recombination shunt resistance (*R*_*sh*_^*recom*^) (reflecting *R*_*sh*_ at *J*_*ph*_ = 0) is increased from 152.0 to 441.8 Ω cm^2^, which may have led to the enhanced *FF*. As the PS templating reduces the internal surface area by occupying the internal space for TiO_2_ nanoparticle-based porous structure, improvement of *R*_*s*_ and *R*_*sh*_^*recom*^ can reasonably be postulated to result from the decrease of interfacial trap sites, which shall be proportional to the internal surface area of TiO_2_ scaffold unless the nature of trap sites at the TiO_2_-perovskite interface are much affected by PS templating. To check whether such explanation works, the correlation of *R*_*s*_ and *R*_*sh*_^*recom*^ with the internal surface area of TiO_2_ layer is plotted in Fig. 5.

The internal surface area of PS-mediated TiO_2_ layer for various PS fractions is calculated based on a simple Monte-Carlo method [46], and the possible overlap between beads is considered rather than assuming the beads as hard-sphere. The random distribution of 200-nm spheres in 10 × 10 × 1 μm^3^ volume is simulated by assuming the probability profile of sphere-to-sphere overlap to show exponential decay, the exponent of which is assumed following the Hertzian model of elastic potential energy for contact of two identical elastic spheres at a given overlap displacement [47]. A previous report on the surface area change by polystyrene particle templating is also given as a more realistic guidance for comparison. In Fig. 5, the experimental PS/TiO_2_ weight ratios are converted to volume ratios from the assumed densities of polystyrene (1.05 g cm^−3^), TiO_2_ (3.91 g cm^−3^) and porosity of mesoscopic TiO_2_ film (68.1%) [15, 48]. By introducing the PS microbeads, the internal surface area is decreased with the improved *R*_*s*_ and (*R*_*sh*_^*recom*^)^−1^. The similar dependence of *R*_*s*_ and (*R*_*sh*_^*recom*^)^−1^ may be from the reduced interfacial traps leading to the decreased resistance of charge transfer or recombination [49]. However, consequently from this simplistic simulation, much drastic variations of *R*_*s*_ and *R*_*sh*_^*recom*^ are correlated with the morphological engineering. This implies that besides the interfacial effects, other factors like the grain size (crystallinity) and thereby the carrier mobility which is affected by the defects or impurities at the grain boundary should be considered to explain the improvement of *R*_*s*_ and *R*_*sh*_^*recom*^, and thereby resultant boost of *η* [7–9, 50].

**Fig. 6:**
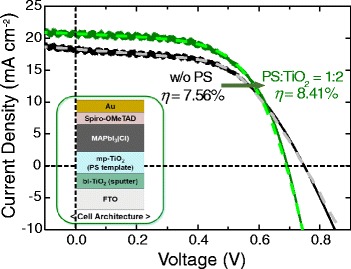
The effect of TiO_2_ blocking layer by sputter deposition on the performance of the perovskite solar cell. *J*-*V* curve (*solid line*) under illumination with the corresponding fitting results (*dashed line*). Photovoltaic parameters are summarized in Tables 3 and 4

**Fig. 7:**
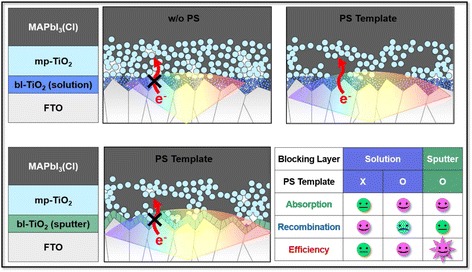
Schematic illustration demonstrating the roles of PS template and TiO_2_ blocking layer on the solar cell performance. The effects of nanostructural engineering by mesoporous TiO_2_ (mp-TiO_2_) and blocking (bl-TiO_2_) layers are illustrated

To further increase *η*, blocking layer deposition method is altered from solution deposition (data in Tables 1 and 2, Fig. 4, and Additional file 1: Figure S6 and S8) to sputter deposition (Tables 3 and 4, Fig. 6, and Additional file 1: Figure S9) to ensure the compactness. Actually, the SEM images in Additional file 1: Figure S10 confirm the porous morphology by the spin-coating and rather compact structure on each FTO grain by sputtering [48, 51–53]. The modified compact blocking layer results in the enhancement of ~0.3 mA cm^−2^ in *J*_sc_, ~30 mV in *V*_oc_, and ~0.7% in *η* (both bare and PS-templated TiO_2_), and consequently, approximately 20% increase of *η* is achieved through the nanostructural control of both blocking and porous layers compared to the bare sample. The compact TiO_2_ hole-blocking layer more effectively inhibits the direct contact between the FTO and the perovskite (preventing the back electron transfer), which is predicted as the origin of charge recombination from the one-diode model. Actually, the modified TiO_2_ blocking layer leads to the decreased *J*_0_ (recombination current) in both bare and PS cases with the slight improvement in *R*_*sh*_^*recom*^, and these are correlated with the improved *V*_oc_ and *FF* as listed in Tables 3 and 4. The performance of the device in this study is not comparable with the state-of-the-art device due to the low *V*_oc_, and the hysteresis problem should be resolved. However, the beneficial optical and electrical properties of perovskite are rationally correlated with the nanostructures to elucidate the origin of the enhanced *J*_sc_. Furthermore, the principal results from the structural engineering in this work will be applicable for various photovoltaic systems utilizing other metal-oxide-based electron-selective contacts and perovskite compositions due to the simplicity of our approach. In addition, the study applying the defect control will further enhance the *V*_oc_ in this device architecture which already shows the promising *J*_sc_ values, potentially improving the device performance even further. As a summary, the effects of intended pore engineering in mesoporous TiO_2_ and the blocking layer are illustrated in Fig. 7.

**Table 3:** The effect of TiO_2_ blocking layer by sputter deposition on the perovskite solar cell performance

Substrate	*J*_sc_ [mA cm^−2^]	*V*_oc_ [V]	*FF* [%]	*η* [%]
w/o PS	18.3	0.749	55.2	7.56
1:2	20.8	0.694	58.3	8.41

Short-circuit current density (*J*_sc_), open-circuit voltage (*V*_oc_), fill factor (*FF*), and power-conversion efficiency (*η*) of perovskite solar cells from w/o PS and PS/TiO_2_ = 1:2. The TiO_2_ blocking layer is deposited by sputtering

**Table 4:** Photovoltaic parameters from the cells with the TiO_2_ blocking layer by the sputter deposition

Substrate	*J*_0_ [mA cm^−2^]	*J*_*ph*_ [mA cm^−2^]	*R*_*s*_ [Ω cm^2^]	*R*_*sh*_ [Ω cm^2^]	*R*_*sh*_^*recom*^ [Ω cm^2^]	*n*
w/o PS	3.53 × 10^−7^ (± 2.93 × 10^−7^)	19.0 (± 0.1)	9.53 (± 0.36)	266.7 (± 2.5)	266.7 (± 2.5)	1.64 (± 0.08)
1:2	2.55 × 10^−3^ (± 0.68 × 10^−3^)	20.8 (± 0.1)	2.18 (± 0.22)	460.9 (± 11.2)	467.8 (± 11.5)	2.98 (± 0.09)

Dark-saturation current density (*J*_0_), photogenerated current density (*J*_*ph*_), series resistance (*R*_*s*_), shunt resistance (*R*_*sh*_), recombination shunt resistance (*R*_*sh*_^*recom*^), and ideality factor (*n*), respectively (AM 1.5 at 100 mW cm^−2^)

## Conclusions

In this work, the mesoscopic TiO_2_ structure was facilely engineered by using a sacrificial template, and the perovskite solar cells were fabricated on nanostructure-controlled scaffolds by systematically examining the concomitant key factors of pore engineering that influence the cell performance. The enhanced efficiency by the enlarged pores was attributed to the effectively infiltrated perovskite grains that provided the beneficial light-harvesting features by the absorption enhancement. The perovskite-TiO_2_ interfacial area was rationally correlated with the internal resistances of solar cell and associated with the charge transfer and recombination. Consequently, the enlarged perovskite grains with the reduced interfacial area contributed together to the internal resistances, changing direction into the efficiency improvement. The leakage current that caused the recombination was successfully resolved through the compact blocking layer, achieving further performance enhancement. We believe that this work suggests a rational nanostructural design of electron-transport layer for high optoelectronic properties in the emerging solar cells.

## Additional file

Additional file 1: Figure S1. SEM images showing the TiO_2_ nanostructures with the PbI_2_ pre-coating and MAPbI_3_(Cl) infiltration into the PS-templated TiO_2_. **Figure S2.** The effect of PS ratio and the concentration of precursor solution on the X-ray diffraction of MAPbI_3_(Cl) perovskite. **Figure S3.** Cross-sectional back scattered electron images exhibiting the MAPbI_3_(Cl) perovskite infiltration in the porous TiO_2_ layer. **Figure S4.** Cross-sectional elemental distributions from energy dispersive X-ray spectroscopy (SEM-EDS) showing the Sn, Ti, O, Pb, and I distributions for different porous TiO_2_ scaffolds. **Figure S5.** Microstructures of MAPbI_3_(Cl) on the TiO_2_ blocking layer. **Figure S6.** Photovoltaic parameters with the average and the standard deviation in each condition. **Figure S7.** Ideal one-diode model for the perovskite solar cell. **Figure S8.** Current density vs. bias under dark and the corresponding fitting results. **Figure S9.** The effect of TiO_2_ blocking layer by sputter deposition on the performance of the perovskite solar cell. **Figure S10.** Morphology comparison by the spin-coating and sputter deposition.
