# Supplementary material for: Tailoring the Mesoscopic TiO2 Layer: Concomitant Parameters for Enabling High-Performance Perovskite Solar Cells
Source: Nanoscale Res Lett. 2017 Jan 19;12:57. doi: 10.1186/s11671-016-1809-7 (PMC5247386; doi:10.1186/s11671-016-1809-7)
Supplement: Additional file 1: Figure S1. — SEM images showing the TiO2 nanostructures with the PbI2 pre-coating and MAPbI3(Cl) infiltration into the PS-templated TiO2. Figure S2. The effect of PS ratio and the concentration of precursor solution on the X-ray diffraction of MAPbI3(Cl) perovskite. Figure S3. Cross-sectional back scattered electron images exhibiting the MAPbI3(Cl) perovskite infiltration in the porous TiO2 layer. Figure S4. Cross-sectional elemental distributions from energy dispersive X-ray spectroscopy (SEM-EDS) showing the Sn, Ti, O, Pb, and I distributions for different porous TiO2 scaffolds. Figure S5. Microstructures of MAPbI3(Cl) on the TiO2 blocking layer. Figure S6. Photovoltaic parameters with the average and the standard deviation in each condition. Figure S7. Ideal one-diode model for the perovskite solar cell. Figure S8. Current density vs. bias under dark and the corresponding fitting results. Figure S9. The effect of TiO2 blocking layer by sputter deposition on the performance of the perovskite solar cell. Figure S10. Morphology comparison by the spin-coating and sputter deposition. [file 11671_2016_1809_MOESM1_ESM.docx]

Supporting Information

**Tailoring the Mesoscopic TiO_2_ Layer: Concomitant Parameters for Enabling High Performance Perovskite Solar Cells**

**Taehyun Hwang,^‡^*^a^* Sangheon Lee,^‡^*^a^* Jinhyun Kim,*^a^* Jaewon Kim,*^a^***

**Chunjoong Kim,*^b^* Byungha Shin,*^c^* and Byungwoo Park**^a^***

*^a^* WCU Hybrid Materials Program, Department of Materials Science and Engineering,

Research Institute of Advanced Materials,

Seoul National University, Seoul 08826, Korea

*^b^* School of Materials Science and Engineering,

Chungnam National University, Daejeon 34134, Korea

*^c^* Department of Materials Science and Engineering,

Korea Advanced Institute of Science and Technology, Daejeon 34141, Korea

^*^ E-mail: byungwoo@snu.ac.kr; Phone: +82-2-880-8319; Fax: +82-2-885-9671.

**^‡^** Two authors contributed equally to this work.

**
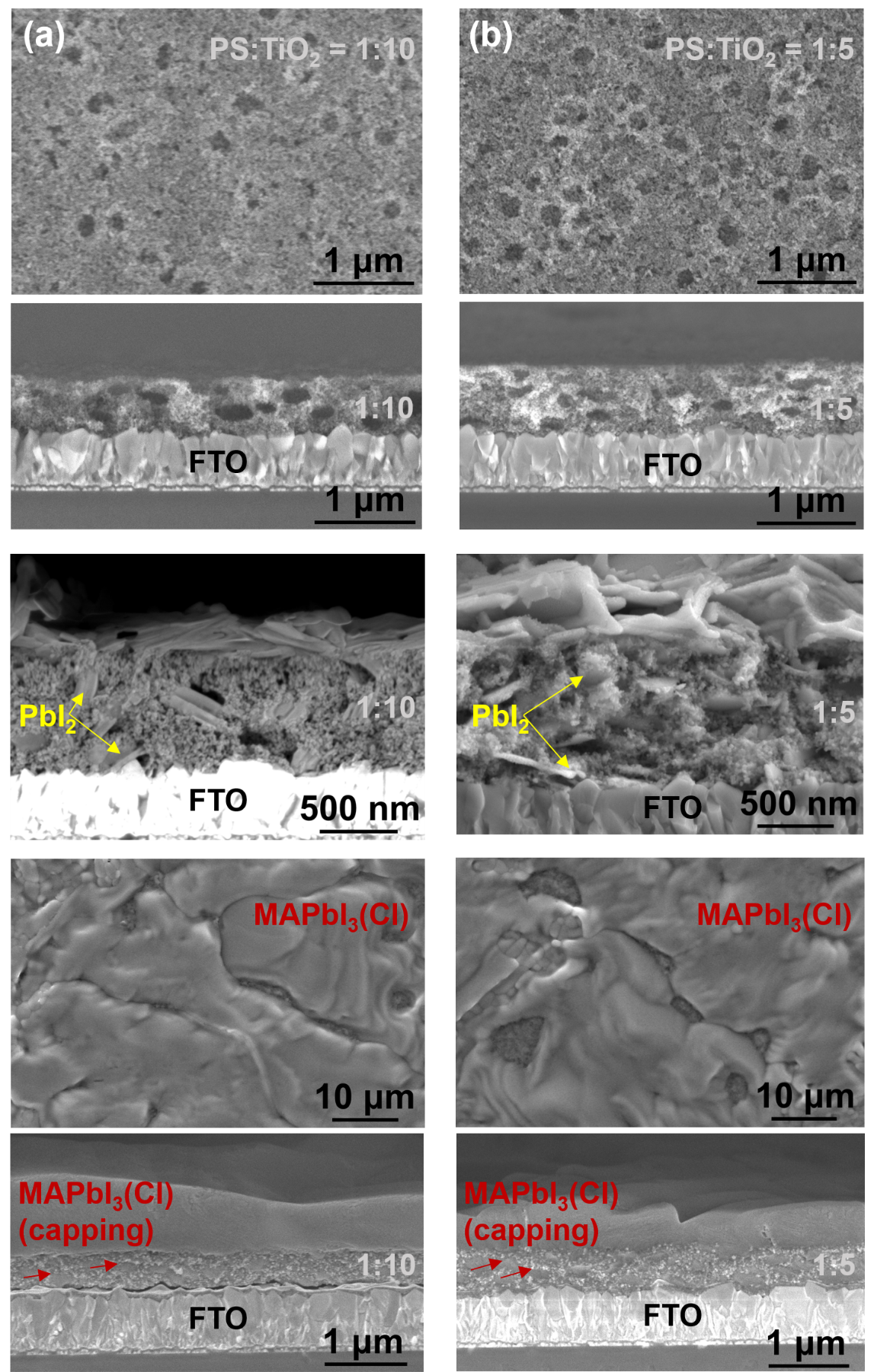
**

**Figure S1. SEM images showing the TiO_2_ nanostructures with the PbI_2_ pre-coating, and MAPbI_3_(Cl) infiltration into the PS-templated TiO_2_.** Porous TiO_2_ layer (top two), TiO_2_ (middle), and MAPbI_3_(Cl) on TiO_2_ (bottom two) with red arrows the MAPbI_3_(Cl). (a) PS:TiO_2_ = 1:10 and (b) PS:TiO_2_ = 1:5.

**Figure S2. The effect of PS ratio and the concentration of precursor solution on the diffraction of MAPbI_3_(Cl) perovskite.** (a) Magnified XRD of MAPbI_3_(Cl) with mixed-halide solution for the three PS cases and for the bare (low precursor than the PS cases). (b) XRD of MAPbI_3_(Cl) with the identical concentration of mixed-halide solution irrespective of the TiO_2_ structures. Details are in the experimental

**
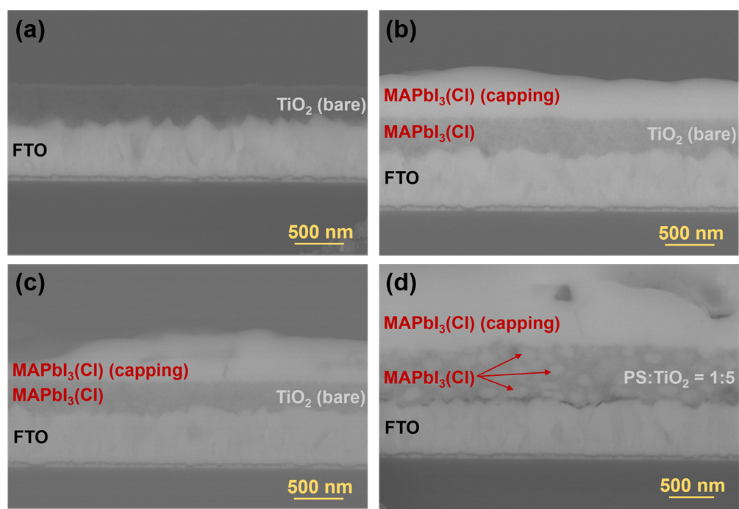
**

**Figure S3. Cross-sectional back scattered electron images exhibiting the MAPbI_3_(Cl) perovskite infiltration in the porous TiO_2_ layer.** (a) Bare TiO_2_, (b) MAPbI_3_(Cl) without PbI_2_ pre-coating on the bare TiO_2_, (c) MAPbI_3_(Cl) with the PbI_2_ and with the PbI_2_ pre-coating on PS:TiO_2_ = 1:5.


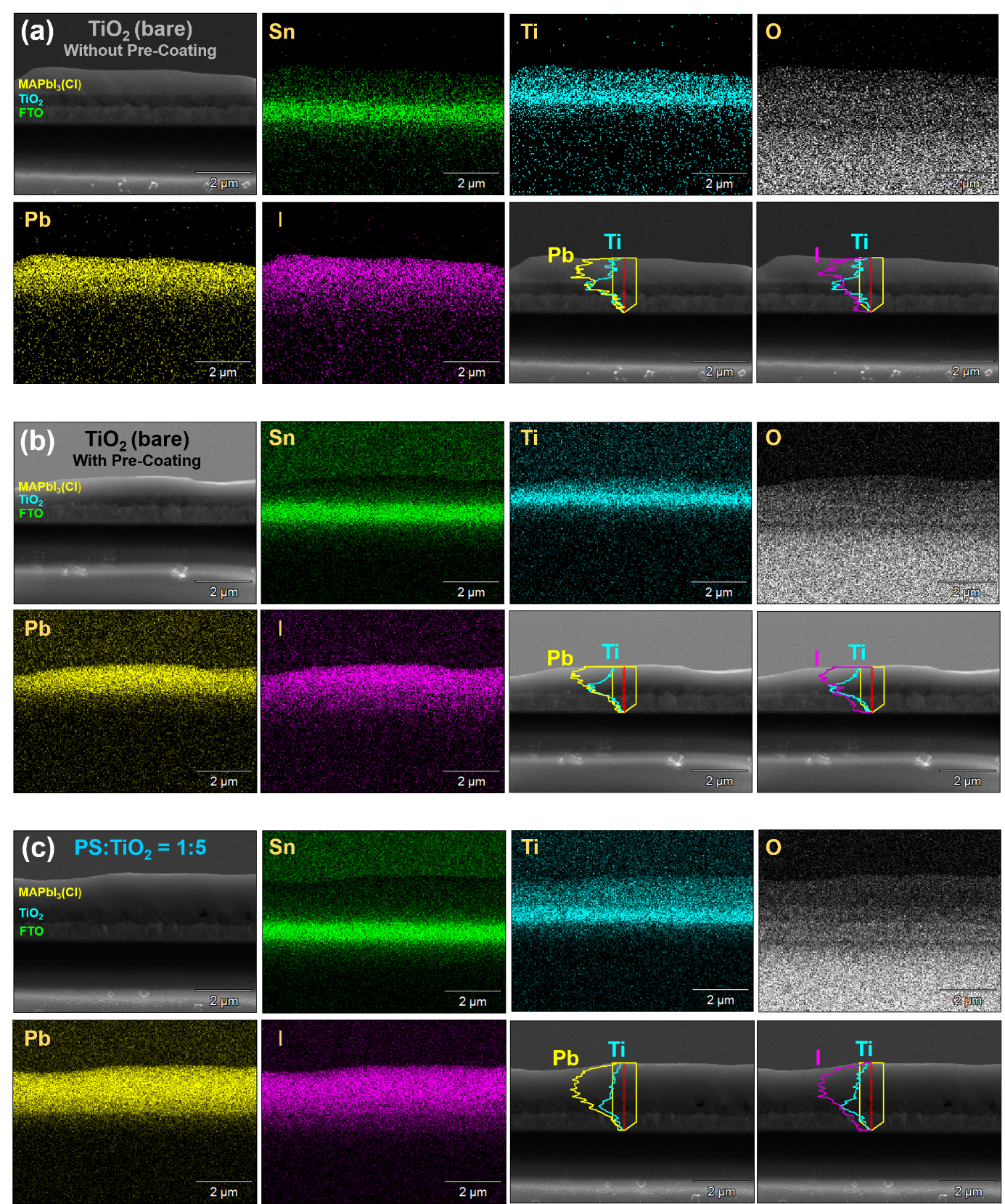


**Figure S4. Cross-sectional elemental distributions from energy dispersive x-ray (SEM-EDS) showing the Sn, Ti, O, Pb, and I distributions for different TiO_2_ scaffolds.** (a) MAPbI_3_(Cl) synthesized without PbI_2_ pre-coating on bare TiO_2_, (b) MAPbI_3_(Cl) with PbI_2_ pre-coating on bare TiO_2_, and (c) MAPbI_3_(Cl) with pre-coating on PS:TiO_2_ = 1:5. Yellow polygon with the middle red line indicates the scan region.

**
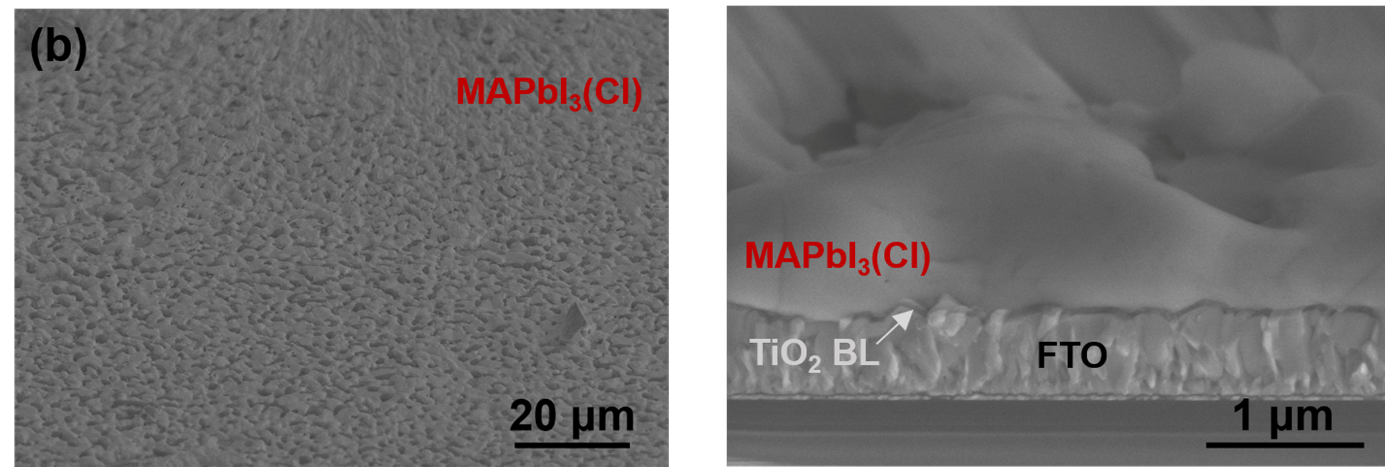
**

**Figure S5. Microstructures of MAPbI_3_(Cl) on the TiO_2_ blocking layer.** (a) XRD of MAPbI_3_(Cl), and (b) the corresponding plan and cross-sectional SEM images of MAPbI_3_(Cl).

**Figure S6. Photovoltaic parameters with the average and the standard deviation in each condition.** (a) *J_sc_*, (b) *V_oc_*, (c) *FF*, and (d) power-conversion efficiency from more than 4 cells in each case. Photovoltaic parameters are summarized in Table 1.

**
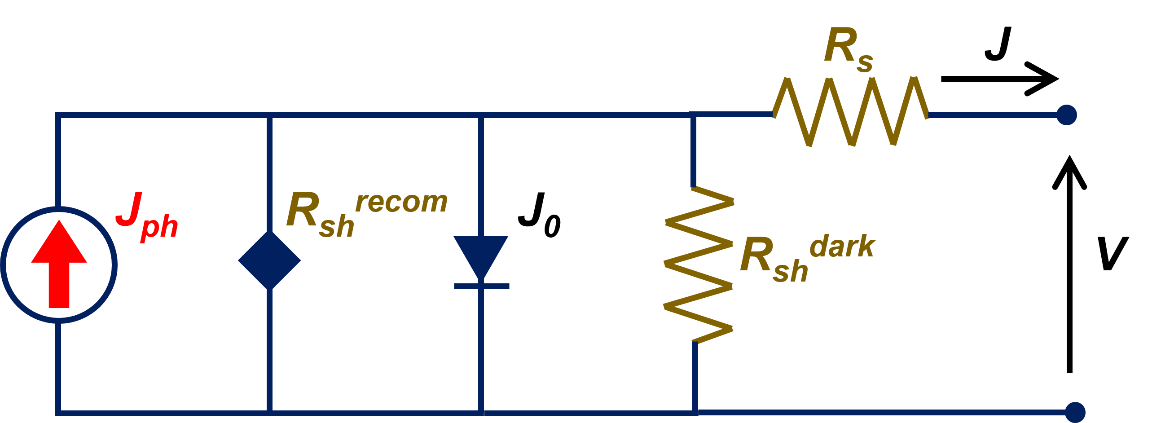
**

**Figure S7. Ideal one-diode model for the perovskite solar cell.**

Ideal one-diode model equation is described as [43,44]:

$$J\left( V \right)=J_{ph}-J_{0}\exp\left[ \frac{e(V+J\left( V \right)R_{s})}{nk_{B}T} \right]-\frac{V+J\left( V \right)R_{s}}{R_{sh}}$$

,

(S1)

where the parameters are photo-generated current density (*J_ph_*), dark saturation current density (*J*_0_), shunt resistance (*R_sh_*), series resistance (*R_s_*), and ideality factor (*n*), respectively. are elementary charge (*e*), Boltzman constant (*k_B_*), and temperature (*T*). The temperature is assumed as 300 K. The recombination shunt resistance *R_sh_^recom^* solar cell operation is derived from the relation:

$$\frac{1}{R_{sh}}=\frac{1}{R_{sh}^{recom}}+\frac{1}{R_{sh}^{dark}}$$

.

.

(S2)

where the *R_sh_^dark^* is derived from the *J*-*V* measured under the dark condition.

**Figure S8. Current density vs. bias under dark and the corresponding fitting results.** TiO_2_, (b) 1:10, (c) 1:5, and (d) 1:2 PS-templated TiO_2_ cases. The scan rate is fixed to the reverse scan of 150 mV s^-1^, which is the same to the solar cell measurement condition.

**Figure S9. The effect of TiO_2_ blocking layer by sputter deposition on the performance of the perovskite solar cell.** *J*-*V* curve under dark condition with the corresponding fitting results (dashed line). Photovoltaic parameters are summarized in Tables 3 and 4.


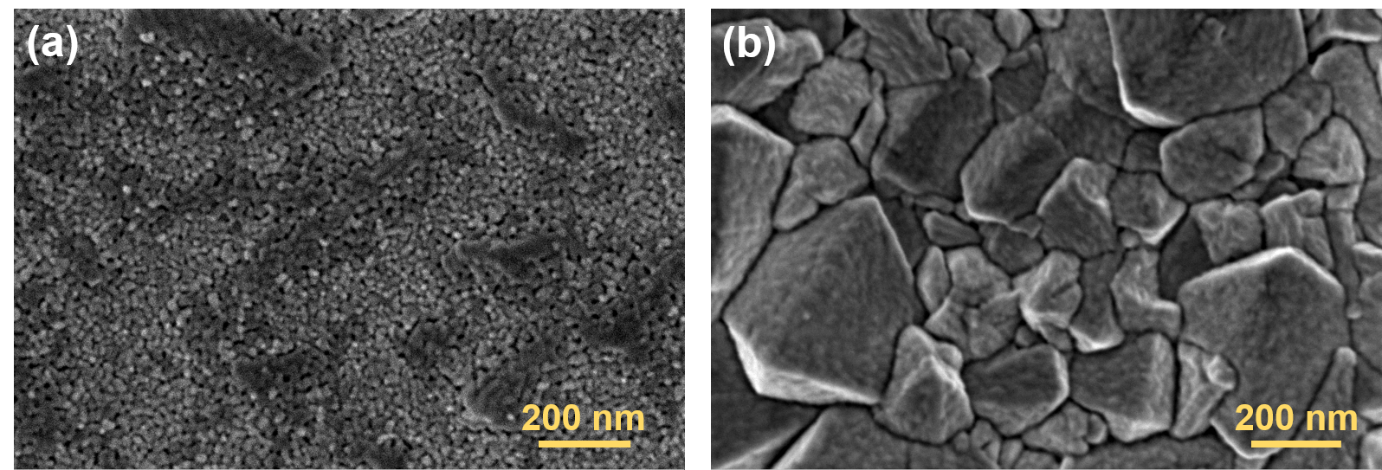


**Figure S10. Morphology comparison by the spin-coating and sputter deposition.** (a) TiO_2_ blocking layer on FTO substrate by spin-coating, and (b) sputter deposition.
